# Pars plana vitrectomy for posterior surface calcification in a silicone intraocular lens in asteroid hyalosis – a report of mistaken identity?

**DOI:** 10.2147/OPTH.S74532

**Published:** 2014-11-13

**Authors:** Paul G Rainsbury, Jonathan Lochhead

**Affiliations:** 1 Eye Unit, Queen Alexandra Hospital, Cosham, Portsmouth, Hants, UK; 2 Eye Unit, St Mary’s Hospital, Newport, Isle of Wight, UK

**Dear editor**

Mehta et al recently reported removal of dystrophic calcification on the posterior surface of a silicone intraocular lens (IOL) in a patient with asteroid hyalosis.^1^ In this case the authors used pars plana vitrectomy (PPV) to successfully remove calcified deposits.

We have recently tried unsuccessfully to use PPV to treat an 86 year old patient with calcification of a silicone IOL in the presence of asteroid hyalosis. We chose to avoid IOL exchange due to a history of Fuchs endothelial dystrophy and glaucoma in the left eye, and a failed corneal graft in a rubeotic eye on the right. Our patient did not have an intact posterior capsule having been treated with Nd:YAG capsulotomy 2 years previously, before the calcification occurred. A 25 gauge pars plana vitrectomy was performed after attempting to remove the deposits unsuccessfully from the posterior surface of the IOL with Nd:YAG laser. It was felt that PPV would be less likely to cause corneal decompensation than IOL exchange. In contrast to the experience of Mehta et al^1^ we found that the vitrector was unable to effectively remove many of the calcified deposits even with high vacuum. Instead the use of microforceps and a silicone tipped flute were required to ‘polish’ the IOL deposits (see Supplementary Video). Although we were able to remove a significant amount of the calcification in this way, the results were ultimately disappointing due to smearing of the deposits which were found to be ‘putty-like’ in consistency. Although there was some improvement in the clarity of the IOL on retroillumination, the patient reported very limited improvement in vision (6/18 preoperatively to 6/12 immediately after surgery), and on transillumination the posterior surface was significantly smudged (Figure 1).

Mehta et al reported that initially their patient underwent an unsuccessful Nd:YAG capsulotomy, however it was not made clear whether the posterior capsule remained intact.^1^ It has previously been suggested that direct contact of the IOL with the vitreous cavity caused by Nd:YAG capsulotomy accelerates the deposition of calcification on silicone IOLs,^2^ and other authors of larger case series of this condition have reported it to be mostly found after initial Nd:YAG capsulotomy,^3^,^4^ although reports of dystrophic calcification in the presence of an intact capsule do exist.^5^ Given our experience, we are unclear whether the photo presented demonstrates typical fibrous capsular opacity, or calcification of the posterior capsule rather than a true case of dystrophic calcification of the IOL optic. We believe this is an important distinction to make since we feel that vitrectomy is only likely to be successful if the posterior capsule is intact.

A similar case of this condition has been published recently by Ullman and Gupta.^5^ They also used vitrectomy to treat dystrophic calcification, but found it to be ineffective at removing established calcification on the posterior IOL surface on more than one occasion. They suggested using prophylactic PPV to treat dystrophic calcification at an early stage before symptoms become established.

We believe that vitrectomy and surgical capsulectomy should be considered as an alternative to Nd:YAG capsulotomy where IOL calcification is not yet established. It is likely that opening a significantly calcified posterior capsule by Nd:YAG capsulotomy in the presence of asteroid hyalosis will lead to significant future IOL dystrophic calcification. In established calcification of a silicone IOL we feel that vitrectomy is of limited value and IOL exchange should remain the standard treatment.

## Figures and Tables

**Figure 1:**
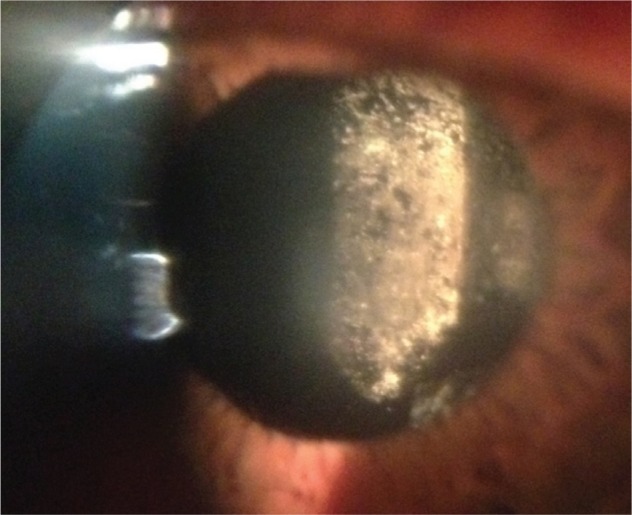
Smudging of posterior surface of IOL on transillumination postoperatively. **Abbreviation:** IOL, intraocular lens.
