# Supplementary material for: Pars plana vitrectomy for posterior surface calcification in a silicone intraocular lens in asteroid hyalosis – a report of mistaken identity?
Source: Clin Ophthalmol. 2014 Nov 13;8:2239–41. doi: 10.2147/OPTH.S74532 (PMC4235510; doi:10.2147/OPTH.S74532)
Supplement: Related texts

# Reply: Authors’ response

**DOI:** 10.2147/OPTH.S74532

**Published:** 2014-11-13

**Authors:** Nitish Mehta, Roger A Goldberg, Chirag P Shah

**Affiliations:** 1 University of Massachusetts Medical School, Worcester, MA, USA; 2 Department of Retina, Ophthalmic Consultants of Boston, Boston, Massachusetts, USA

We appreciate Drs Rainsbury and Lochhead sharing their experience and insight into the treatment of dystrophic calcification of silicone intraocular lenses (IOL). As they point out, it intuitively makes sense that the presence or absence of a posterior capsule changes the natural history and management options of this pathologic calcification.

In our case,^1^ because of the poor visual potential in the fellow eye from retinal scarring and atrophy secondary to age-related macular degeneration, we were reluctant to proceed with IOL exchange, though would have if necessary. The patient had already received two prior unsuccessful attempts with the Nd:YAG laser to remove the dystrophic material. During the vitrectomy, no clean surgical plane developed, and a modest degree of mechanical rubbing of the vitrectomy port against the lens optic using a high vacuum with a low cut rate was required to clear the central axis. The material appeared to be adherent directly to the posterior surface of the lens optic, and not to any residual posterior capsule, suggesting the posterior capsule was absent centrally. Had the posterior capsule been present, we suspect it would have been easier to remove the dystrophic material.

In the setting of monocular patients (as in our case) or those with compromised capsular support (as in the case described by Drs Rainsbury and Lochhead), we believe it is reasonable to attempt vitrectomy prior to IOL exchange in these cases of dystrophic calcification. Though vitrectomy might not be successful in every case, removing the vitreous alone may make subsequent IOL exchange safer, and prevent recurrence of dystrophic calcification.

## References

[1] Mehta N, Goldberg RA, Shah CP (2014). Treatment of dystrophic calcification on a silicone intraocular lens with pars plana vitrectomy. Clin Ophthalmol.
